# Valorization of Carrot Pomace into Mycelium-Based Paper for Packaging Applications

**DOI:** 10.1021/acsomega.6c00730

**Published:** 2026-05-18

**Authors:** S. Najmeh Mousavi, Naba Kumar Kalita, Sunil Kumar Lindstrom Ramamoorthy, Minna Hakkarainen, Akram Zamani

**Affiliations:** † Swedish Center for Resource Recovery, 1802 University of Borås, Borås 501 90, Sweden; ‡ Natural Resources Research Institute, University of Minnesota, Duluth, Minnesota 55811, United States; § Department of Fiber and Polymer Technology, 7655 KTH Royal Institute of Technology, Stockholm 100 44, Sweden

## Abstract

Mycelium-based materials are promising environmentally friendly alternatives to synthetic materials. Utilizing industrial fruit and vegetable waste as a low-cost substrate presents a potential pathway for large-scale fungal biomass (FB) production, thereby facilitating the production of mycelium-based materials. In this study, carrot pomace (CP) was used as a substrate for cultivating two filamentous fungi, *Rhizopus delemar* and *Aspergillus oryzae* (AO), in bench-scale bioreactors. Harvested solids containing mycelium and CP residues were processed into hybrid paper, mycelium-based paper (MBP), through a wet-laid process. To obtain flexible paper, MBP was then post-treated with glycerol as a plasticizer. Scanning electron microscopy images of the recovered solids showed an interconnected thin microfibrillar structure in AO, whereas *Rhizopus delemar* demonstrated shorter microfibers with larger diameters. The cross-sectional images of AO-MBP showed a more entangled network structure, with a smaller average pore size (36 μm) compared to RD-MBP (45.7 μm), indicating a more compact microstructure. The tensile strength of AO-MBP was 49 MPa, while RD-MBP displayed a lower tensile strength of 32 MPa. Post-treatment with glycerol reduced average pore size and tensile strength; however, elongation at break was enhanced by 60% for both AO-MBP and RD-MBP compared to untreated samples, resulting in a flexible material suitable for use as wrapping paper. The mechanical properties of MBP were comparable to those of commercial paper products, according to the material property charts. This paves the way for a fungal biorefinery concept for valorizing CP to novel paper-like products with potential applications in packaging.

## Graphical abstract

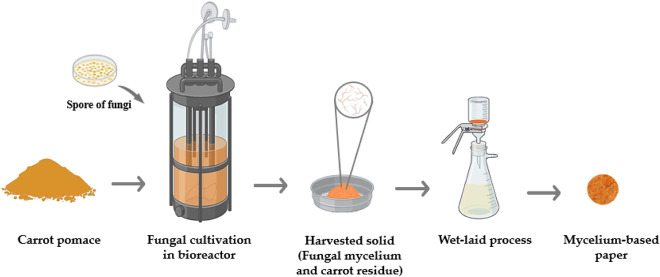

## 1 Introduction

Developing new materials from industrial byproducts is essential to address global environmental and economic challenges and moving toward achieving net-zero waste. The European juice industry generates substantial solid waste annually, estimated at approximately 5.5 billion kilograms.^1^ Valorizing this waste into high-value products and establishing innovative biobased alternatives are promising approaches, particularly given the fiber-rich nature of these residues.

Carrot pomace (CP), a byproduct of the carrot juice industry, contains a substantial fraction of structural polysaccharides typical of plant cell walls. On a dry mass basis, it consists of approximately 10–28% cellulose, 5–20% hemicellulose (composed of diverse heteropolysaccharides, including arabinogalactans and xyloglucans), lignin (2.5–7.7%) and 2–8% pectin (rich in galacturonic acid domains).^2–5^ These components have been utilized in material development, including the production of nanocellulose films extracted from carrot pulp^6,7^ or as a filler in chitosan films.^8^ These structural polysaccharides are generally not readily susceptible to microbial conversion unless they are pretreated and hydrolyzed into smaller, more accessible carbohydrate fractions. Beyond the fibrous components, CP also contains soluble sugars and other essential nutrients that support microbial growth. Owing to the presence of fermentable sugars, CP represents a cost-effective and renewable substrate for microbial cultivation^9–12^ and for the development of biomaterials, such as bacterial cellulose. Bacterial cellulose is typically produced in static or submerged cultures using cellulose-producing bacterial strains. When waste substrates are used, pretreatments are often required to release soluble sugars. Cultivation in static systems generally takes around 7 days and results in a pellicle composed of pure cellulose nanofibers with high potential for applications such as in the medical field.^13,14^ Filamentous fungi represent another group of microorganisms that can be cultivated using fermentable sugars from fruit and vegetable wastes.

In our previous study, we showed that the sugar-rich liquid of CP can be separated from the fibrous structure and used for fungal cultivation, resulting in FB with potential food or feed applications.^15^ Filamentous fungi grow as mycelium, a network of hyphae with diameters ranging from 1 to 30 μm, spreading throughout the substrate and binding the substrate particles together, effectively acting as an adhesive in the substrate structure.^16^ The process is generally mild and can be performed under controlled conditions to optimize yield.^17^ Mycelium-based materials can serve as biobased alternatives to synthetic materials for various applications including textiles, packaging, and construction.^18,19^

Solid-state fermentation and liquid or submerged fermentation are two primary methods used for mycelial production. In solid-state fermentation, the mycelium grows on a solid substrate, such as inexpensive agricultural residues, whereas liquid or submerged fermentation involves the growth of microorganisms, such as fungi, in a liquid medium containing nutrients. Solid-state fermentation is commonly used to produce bulk mycelium-based composites from lignocellulosic waste that are suitable for construction applications.^20,21^ Extensive research has been conducted to fabricate pure mycelium sheets from different fungal strains that are cultivated in solid-state cultures containing synthetic media, such as potato dextrose agar (PDA) and potato dextrose broth (PDB).^22–25^ Furthermore, various fungal strains have been cultivated via submerged fermentation using substrates such as PDA, and yeast extract peptone dextrose (YPD) has been employed for growing *Pleurotus ostreatus* in submerged cultivation.^26–28^ FB from submerged cultivation of *Rhizopus delemar* using bread waste as a substrate has been tested for different applications, including the production of textile filaments, leather-like materials, and films for packaging purposes.^29–31^

Hybrid-mycelium sheets, made of a combination of mycelium and cellulosic components, have gained attention because of their ability to address the strength limitations of pure mycelium sheets.^23,25^ Attias et al.^32^ introduced hybrid materials by integrating nano cellulose into a synthetic medium used for the cultivation of *Trametes ochracea* for packaging applications. In another study, the cell wall materials of three strains of fungi were blended with bleached softwood kraft and hemp fibers at different mass ratios. Subsequently, the blended materials underwent disintegration and were used to produce sheets with dense fibrillary networks via a wet-laid process. These sheets, known as Mycocel biopolymers, have been examined for packaging, filtration, and hygiene products.^33^ The intrinsic brittleness and inflexibility of the mycelium-based materials limit their applications.^30^ However, it is noteworthy that glycerol and hot-press treatments can improve the mechanical properties of mycelium films, as demonstrated in the research conducted by Shao et al.^18^

Several studies have focused on developing mycelium-based materials by inoculating fungal species on lignocellulosic waste streams from agricultural or forestry sources, such as corn husks or sawdust.^34^ Our previous studies focused on use of CP as a substrate for mycelium production. A nutrient-rich liquid was extracted through enzymatic pretreatment of the CP. The liquid was used as a substrate to cultivate *Rhizopus delemar*.^15^ Subsequently, a hybrid mycelium-based paper was produced by combining the FB with the remaining cellulosic fraction of CP, and demonstrated for dye removal from wastewater.^35^

The selection of safe microorganisms is crucial in the development of processes for the production of biotechnology-based products as it facilitates faster regulatory approvals before upscaling the processes. Therefore, this study focused on nontoxic microorganisms classified under Biosafety Level 1 (BSL-1). Two BSL-1 strains, *Rhizopus delemar* and *Aspergillus oryzae*, were selected from two major groups of filamentous fungi i.e., mucoromycetes and ascomycetes.^36^ These fungi were chosen for their high growth rates and ability to grow on different low-quality substrates, such as fruit and vegetable waste.^37,38^

The innovative aspect of this study lies in the utilization of the entire CP instead of a nutrient-rich liquid medium. The recovered solid, containing FB and the cellulosic fraction of CP, was subsequently employed to produce mycelium-based papers (MBP). Glycerol, a cost-effective, biodegradable, and nontoxic plasticizer,^39^ was employed to enhance flexibility by disrupting intermolecular polymer interactions (particularly hydrogen bonding) and forming new polymer–glycerol interactions, while hot-press was applied as a postprocessing step to increase the density and improve the mechanical properties, durability, and barrier performance by reducing average pore size. The resulting mycelium-based paper (MBP) was evaluated for its potential in packaging applications. Figure 1 shows a graphical representation of this study.

**Figure 1:**
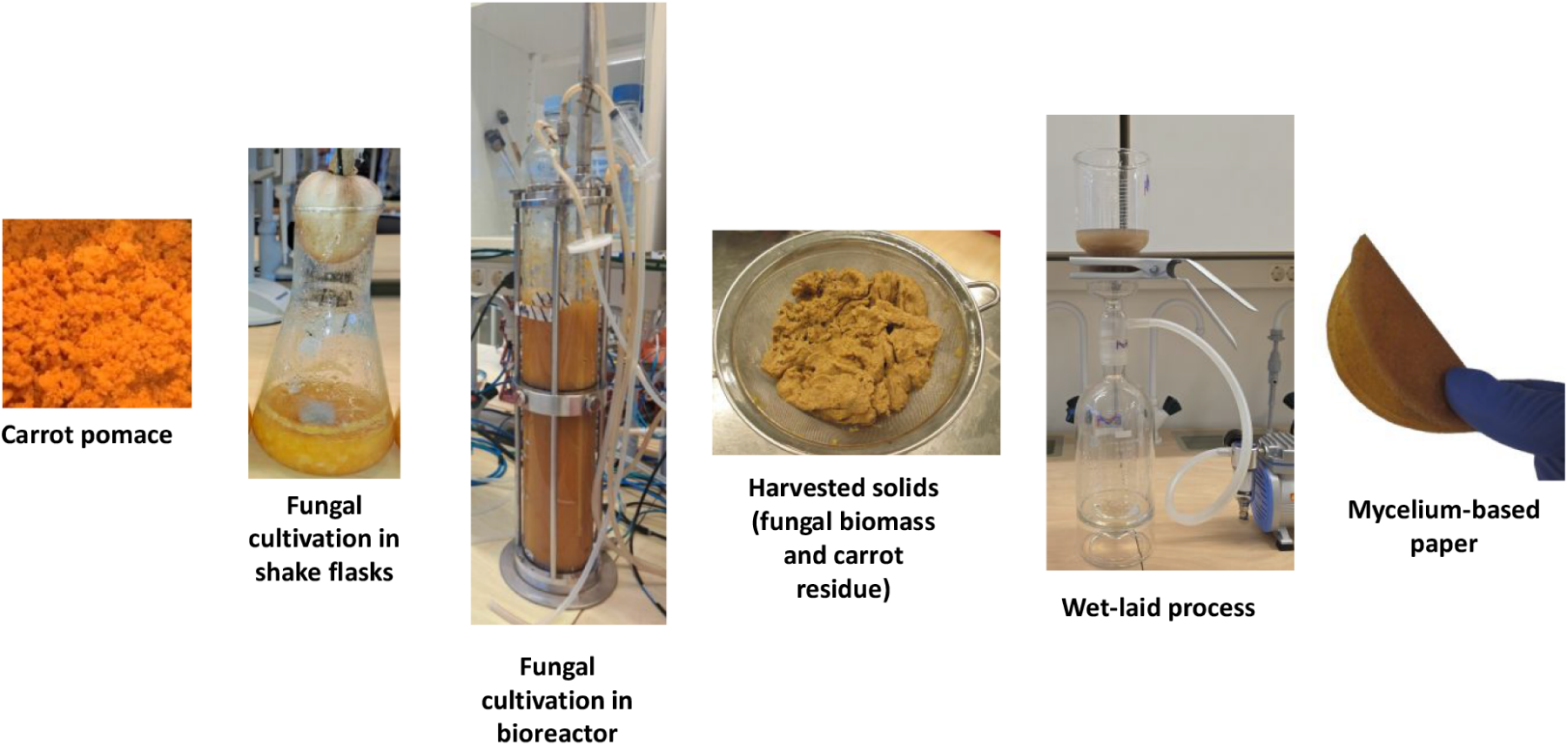
Schematic of the process from carrot waste to mycelium-based paper. Carrot pomace is cultivated with filamentous fungi in shake flask and bubble-column bioreactor. The harvested solids are then ground, and the resulting suspension is converted into mycelium-based paper through a wet-laying process.

## 2 Materials and Methods

### 2.1 Substrate and Fungal Strains

Carrot pomace was kindly provided by Herrljunga Cider AB (Sweden) and stored at −18 °C until use. The fungus *R. delemar*, CBS 145.940 (Centraalbureau voor Schimmelcultures, Westerdijk Institute, Utrecht, The Netherlands) and *A. oryzae* CBS 819.72 (Centraalbureau voor Schimmelcultures, Utrecht, The Netherlands) were used in this study. Agar, hydrochloric acid (HCl), sodium hydroxide (NaOH), peptone, glucose, potato infusion, invertase from Baker’s yeast (*S. cerevisiae*), and glycerol were purchased from Sigma-Aldrich (St. Louis, MO, USA).

Agar plates inoculated with spores of *R. delemar* were prepared according to our previous work.^15^ *A. oryzae* was maintained on Potato Dextrose Agar (PDA) plates containing 4 g/L potato infusion, 20 g/L glucose, and 15 g/L agar at 30 °C for 3 days. After incubation, the plates containing the grown fungi were stored at 4 °C until use.^40^ The abbreviations used are listed in Table 1.

**Table 1:** List of Abbreviations Used in This Article

Abbreviation	Definitions
CP	Carrot Pomace
FB	Fungal Biomass
MBP	Mycelium-Based Papers
PDA	Potato Dextrose Agar
PDB	Potato Dextrose Broth
AO	*Aspergillus oryzae*
RD	*Rhizopus delemar*

### 2.2 Fungal Cultivation

#### 2.2.1 Submerged Cultivation in Shaking Flasks and Bench-Scale Bioreactors

To prepare the substrate for fungal cultivation, CP was mixed with water to obtain a suspension with a dry content of 4%. Before cultivation of *R. delemar*, invertase was added to the suspension to hydrolyze sucrose into glucose and fructose. This pretreatment is essential because sucrose is not consumable by *R. delemar*, as reported in our previous study.^15^ However, *A. oryzae* can consume sucrose as a carbon source;^41^ therefore pretreatment is not required for this fungus.

Fungal cultivation was conducted in 250 mL Erlenmeyer flasks sealed with cotton plugs, each containing 200 mL of CP suspension (4%). The pH was adjusted to 5.5 before sterilization in an autoclave (VX-95, Systec, Linden, Germany) at 121 °C for 20 min. The flasks were then inoculated with 4 mL spore suspension of either *R. delemar* or *A. oryzae*. Fungal growth was performed at 35 °C in a water bath shaker (Grant Instruments Ltd., Cambridge, UK) at 100 rpm for 72 h, and samples were taken every 24 h to analyze sugar and ethanol contents. Finally, the solid fraction containing the FB and carrot residues was harvested using a kitchen sieve, washed with distilled water, and dried in an oven at 70 °C. All experiments were performed in duplicate.

Fungal cultivation was upscaled to bench-scale 4.5 L bubble column glass bioreactors (Belach Bioteknik AB, Skogås, Sweden) with a working volume of 3.5 L. A suspension of invertase pretreated CP and untreated CP in water was prepared to cultivate *R. delemar* and *A. oryzae*, respectively. The substrate solid loading used for both fungal strains was 4 wt %. The pH of the suspensions was adjusted to 5.5. The bioreactors were filled with the suspensions, sterilized in an autoclave (VX-95, Systec, Linden, Germany) at 121 °C for 20 min. After cooling, the inoculation was done with 70 mL of spore suspensions (0.02 mL/mL of the substrate). Fungal cultivation was conducted at an aeration rate of 5 vvm (volume of air per volume of liquid per minute) at 35 °C for 72 h. Throughout the cultivation, the pH was monitored and readjusted manually to 5.5 when needed, by addition of 2 M NaOH. Liquid samples were taken every 24 h to analyze sugars and ethanol contents. At the end of the cultivations, the solids, containing the FB of *R. delemar* or *A. oryzae* mixed with CP residues, were harvested by filtration using a kitchen sieve and washed with water. Samples were taken to determine the dry mass content, and the rest was stored in wet form at −18 °C until use.

#### 2.2.2 Morphology of the Harvested Solids

The morphology of the harvested solids, including fungal biomass and CP residue, was analyzed by scanning electron microscopy (SEM). The harvested solid was promptly frozen in liquid nitrogen to preserve its morphological configuration and then freeze-dried. Images were captured by an ultrahigh-resolution FE-SEM (Hitachi S4800). The samples were coated with a 2 nm Pd/Pt layer, and images were taken at a magnification of 1,000× with an accelerating voltage of 3 kV.

### 2.3 Preparation of Mycelium-Based Paper

Before using the harvested solids for the development of mycelium-based paper, the material was sterilized in an autoclave (VX-95; Systec, Linden, Germany) to stop any fungal cell activity. Subsequently, 1% suspension of the recovered material was prepared in distilled water. To ensure a homogeneous suspension, it was passed through a disk mill grinder supermasscolloider (MKCA6-5J, Masuko Sangyo, Kawaguchi, Japan). The grinding process involved five cycles with standard silicon carbide grinding stones (MKE 46) at a rotational speed of 2700 rpm. The gap between the stones was adjusted to 50 μm (open gap). The ground suspension was stored at 4 °C until use.

Mycelium-based paper (MBP) was prepared by a wet-laid process using a vacuum funnel filtration setup, resulting in the formation of circular sheets (7 cm in diameter). The process employed a membrane with a 30 μm pore size (Spectra Mesh woven filters, Nylon, Thermo Fisher Scientific, USA). The ground suspension was diluted to a final grammage of 120 g/m^2^ after drying MBP. The final grammage was set to match commercial packaging paper manufactured from virgin fiber through the kraft papermaking process, which is commonly used in the production of commercial paper shopping bags on the market.

During the filtration process, water removal facilitated both the physical entanglement and hydrogen bonding of the mycelium and −CP particles, resulting in the formation of a cohesive network that formed the structural basis of MBP. Polysaccharides present in the mycelial hyphal matrix play a crucial role in promoting hydrogen bonding, which is critical for the aggregation and stability of the network.^42^ The filtration time was recorded, and the fraction of the material that passed through the membrane was measured. Wet mycelium-based paper was placed between two blotting papers (Ahlstrom-Munksjö Falun AB, Sweden) to remove excess water. Finally, to prevent shrinkage of the sheets during drying, they were fixed between flat Plexiglas sheets and rings and placed under a fume hood at room temperature for 24 h to dry.

#### 2.3.1 Post-Treatments

Glycerol was added as a plasticizer to enhance the flexibility of MBP. A 20% v/v glycerol solution was prepared in water, and the dried MBP was then immersed in 500 mL of glycerol solution for 1 h. Afterward, the wet MBP was dewatered with blotting paper and dried at room temperature (21 °C) using the same setup detailed in Section 2.3, to prevent shrinkage. In another post-treatment, the MBP was pressed at 80 kN and 100 °C for 2 min, following the procedure outlined in ref 18. The objective of this treatment was to provide conditions similar to those in the calendaring process in papermaking.

#### 2.3.2 Morphology and Physical Properties of MBP

The thickness (d) of the MBP was determined using a digital micrometer (Mitutoyo, Japan). Grammage refers to the weight of the paper expressed as grams per square meter (g/m^2^). This term is commonly used in the pulp and paper industry and is an important factor in determining the quality and application of different types of paper. Grammage was calculated as the sample weight divided by the area of 7 cm diameter circles. The density (kg/m^3^) was calculated by dividing the weight of paper by its thickness (m) and given area (m^2^). The volume was calculated by multiplying the MBP thickness (m) by the area (m2). The gas permeability of the MBP was examined using PSM 165 porosimeter (Topas GmbH, PSM 165, Germany) to determine the pressure drop of air across the MBP. Air was injected into the sample holder with a cross-section of 16 mm under a pressure of 2000 mb and air flow of 70 L/min. Measurements were conducted for both the dry and wet MBP. Topor (Topas GmbH, with a surface tension of 16.0 mN m^–1^ and density of 1.9 gmL^–1^, both at 25 ^◦^C) was used as the wetting fluid.^43^ All tests were performed in triplicates. It should be noted that the instrument provides an apparent (equivalent) average pore size derived from air permeability using porous media models, rather than a direct geometric measurement. Therefore, it is treated as a model-based indicator of gas transport behavior. Colorimetry (CR-10 plus, Konica Minolta, Inc., Japan) was used to determine the MBP color. The data were measured based on CIE Lab color parameters in terms of L*, a*, and b*. The mean values were calculated from five measurements taken from different sections.

#### 2.3.3 Mechanical Tests and Material Comparison Using Ashby’s Plots

The tensile properties of MBP were assessed following ISO 527-2 (2012), which is widely used for polymer testing. While ASTM D638 is another recognized standard, both methods share similarities in specimen geometry, gauge length, and testing speed, ensuring comparable mechanical property measurements. Literature suggests that tensile results from ISO 527-2 and ASTM D638 are generally consistent for flexible polymeric materials. Given the alignment of our test parameters with those in ASTM D638, we expect the results to be reliable and comparable. To determine the mechanical properties of MBP, dog-bone specimens were cut according to ISO 527-2 (2012) using a press knife (Elastocon, Sweden). The samples were then subjected to mechanical property analysis using a tensile testing machine (H10KT, Tinius Olsen, USA) equipped with a 100 N load cell. The gauge length and speed were set at 26 mm and 5 mm/min, respectively. The tensile strength (MPa) and elongation at break (%) were determined using the QMat software (Tinius Olsen, USA), while Young’s modulus (E) was calculated from the slope of the stress–strain curve in the linear region between 0.1–0.5% strain.

To assess the position of MBP in comparison to commercially available materials, Ashby’s bubble chart software was utilized to visually represent the material properties based on one physical and one mechanical behavior. To perform the material comparison, the tensile strength was plotted against the density using the level 3 material database of the software. The graph was created using Granta Edupack 2021 R2 version: 21.2.0 (Ansys Inc. USA).

#### 2.3.4 Thermogravimetric Analysis (TGA)

TGA analysis was conducted using a Q500 TA Instruments apparatus (Waters LLC, USA) to determine the thermal decomposition characteristics of the MBP. Approximately 10 mg of each sample was heated in a furnace from room temperature to 600 °C at a rate of 10 °C/min under a nitrogen atmosphere. The experiment was performed in duplicate, and graphs depicting the percentage of weight versus temperature were generated.

#### 2.3.5 Differential Scanning Calorimetry (DSC)

The thermal behavior of MBP was studied using a TA Instruments Q200 (New Castle, DE, USA). Approximately 5–10 mg of each sample, with a moisture content of 7%, was placed in Tzero aluminum pans and sealed with lids. Thermograms were obtained in the range of −20 to 400 °C, with an initial equilibration step at −20 °C for 1 min. The temperature was increased at a rate of 5 °C min^–1^ under a nitrogen atmosphere. The glass transition temperature (*T*_g_) was determined from the heat flow.

#### 2.3.6 Fourier Transform Infrared (FTIR) Spectroscopy

A Fourier transform infrared (FTIR) spectrometer (Nicolet iS10, Thermo Fisher Scientific, USA) operated in transmission mode was used to analyze the chemical composition of MBP using OMNIC 4.1 software. The paper samples were analyzed directly without ATR accessories or KBr pellet preparation. Prior to analysis, the samples were kept in a desiccator for 24 h to minimize excess moisture, corresponding to a moisture content of approximately 7% during measurement. Spectra were recorded over the range of 500–4000 cm^–1^ with 32 scans at a resolution of 4 cm^–1^. A background spectrum was collected and applied to correct atmospheric and instrumental contributions. The resulting spectra were normalized and used to identify functional groups and molecular interactions in MBP

#### 2.3.7 Water Contact Angle (WCA)

To assess the water-repellence properties of MBP, a water contact angle test was conducted using an optical tensiometer and One Attention software (Biolin Scientific, Espoo, Finland). A 4 μL water droplet was dispensed onto the surface of a 2 × 2 cm^2^ MBP sample, and the images were captured at room temperature.

#### 2.3.8 Statistical Analysis

Statistical analysis was conducted using analysis of variance (ANOVA) and post hoc Tukey test. p-values smaller than 0.05 were considered statistically significant.

## 3 Results and Discussion

The whole CP was explored as a substrate for fungal cultivation using submerged cultivation processes. Two fungal strains were selected, and their growth efficiency was monitored throughout the cultivation process. The material recovered from submerged cultivation was subjected to mechanical homogenization by ultrafine grinding and developed into a hybrid paper. The resulting products were characterized by assessing their morphological, physical, mechanical, thermal, and chemical properties.

### 3.1 Fungal Cultivation

#### 3.1.1 Submerged Cultivation

Submerged cultivation was initially performed in shake flask and then scaled up to bench bioreactors (4.5 L) (Figure S2). In the shake flask experiments, the concentrations of total recovered solids, comprising a mixture of mycelium and CP residues, after cultivation of *R. delemar* and *A. oryzae*, were 8.19 and 8.85 g/L, respectively (Table 2). However, this concentration decreased to 13.44 for *R. delemar* and 8.5 g/L for *A. oryzae* when cultivated in 4 L reactors. This reduction may be attributed to the increased passage of solid material through the sieve during the harvesting process due to differences in the pore sizes of the small and large sieves used for harvesting the solids from shake flasks and the reactors, respectively. The dry mass of the remaining soluble solids after the cultivation of *R. delemar* was higher compared to *A. oryzae*. This indicates a higher ability of *A. oryzae* to consume the available nutrients in the medium, resulting in higher biomass production, which is in line with previous studies.^44,45^

**Table 2:** Result of Fungal Cultivation in Shaking Flasks and Bench-Scale Bioreactors

**Scale of fermentation**	**Type of fungus**	**Total recovered solids** (g/L)	**DM of soluble solid after cultivation (%)**
Shake flask	*R. delemar*	8.19 ± 0.39	1.35 ± 0.02
	*A. oryzae*	8.58 ± 0.23	0.88± 0.02
4L Reactor	*R. delemar*	13.44 ± 0.85	1.83 ± 0.04
	*A. oryzae*	12.68 ± 0.57	1.42 ± 0.32

To monitor nutrient consumption and metabolite formation during fungal growth, liquid samples taken from the shake flasks and bioreactors were analyzed using HPLC, and the concentrations of glucose, sugar mix, and ethanol were measured (Figure 2). The sugar mix included monomeric sugars other than glucose such as xylose, fructose, galactose, and arabinose. During cultivation in shake flasks, a major fraction of the available glucose (13.2 and 11.0 g/L) was consumed by both fungal strains within 24 h, followed by nearly complete depletion of glucose after 72 h (Figure 2). The initial concentration of sugar mix was 11.8 and 12.8 g/L in flasks used for the cultivation of *Aspergillus oryzae* (AO) and *Rhizopus delemar* (RD), respectively, which decreased to 0.2 and 0.6 g/L, respectively (Figure 2b). This pattern reflects the selective sugar utilization characteristic of fungi, where glucose serves as the primary carbon source and is rapidly consumed, while other sugars are metabolized more gradually.^46^ During cultivation, ethanol was also formed as the main metabolite and the concentration increased to 4.0 and 7.2 g/L for AO and RD, respectively, within 72 h (Figure 2c). A similar pattern was reported for the growth of RD-on the liquid fraction of CP.^15^ The higher level of ethanol production by RD agrees with the lower concentration of recovered solids for RD fungus compared to AO.

**Figure 2:**
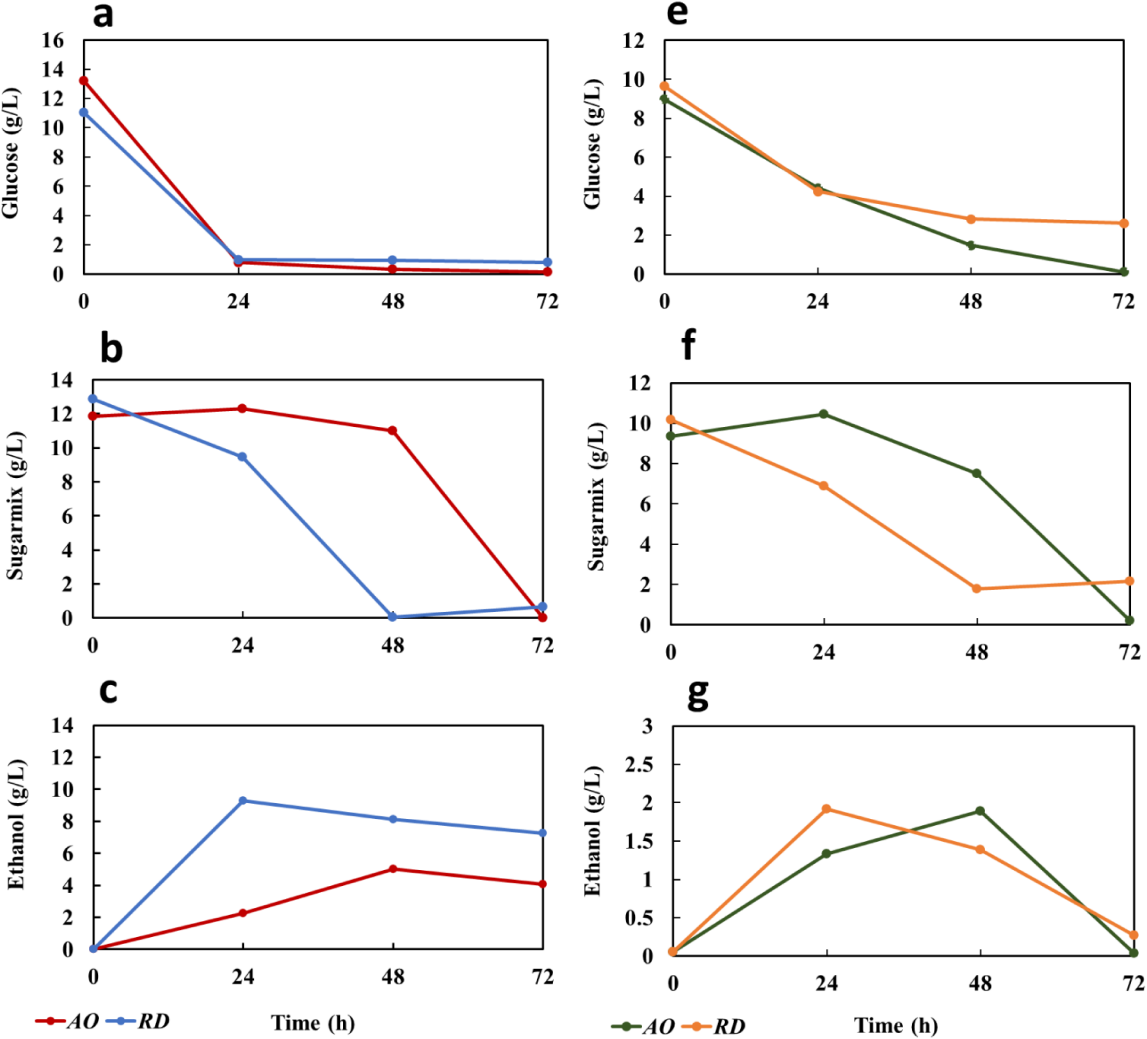
Glucose, sugar mix, and ethanol concentration profiles during cultivation of *A. oryzae* (AO) and *R. delemar* (RD), on CP in shake flasks (a, b and c) and in a 4.5 L bench-scale bubble column bioreactor (d, e and f) during 72 h. The sugar mix included monomeric sugars other than glucose, such as xylose, fructose, galactose, and arabinose. Glucose concentration (a, d), sugar mix concentration (b, e), and ethanol concentration (c, f). The standard deviations were 0.2–3.8% for bioreactors and 0.007–0.9% for shake flasks.

In the bioreactors, AO consumed 99% of the available glucose within 72 h, whereas RD retained 3% of the initial glucose after the same period (Figure 2d). Regarding the sugar mix concentration, some sugars are freely available from the initiation of the cultivation process, facilitating immediate fungal consumption.^47^ Subsequently, the fungus secretes enzymes capable of hydrolyzing the remaining substrate into free sugars. For AO, after 48 h, the rate of enzymatic hydrolysis appeared to exceed the rate of sugar consumption, resulting in an increase in the sugar mix concentration. Conversely, RD exhibited a comparatively lower hydrolysis rate, therefore, no significant increase in sugar concentration during the cultivation period was observed (Figure 2e). Notably, enzymatic pretreatment with invertase was not required before the AO cultivation due to *Aspergillus oryzae* metabolic flexibility and adaptability. This enables the utilization of various sugars by producing diverse enzymes including amylases, invertases, and glucosidases. These enzymes facilitate the hydrolysis of complex sugars into simpler forms that can be consumed for energy generation and cell growth.^41^ A similar pattern was reported for the growth of the *R. delemar* fungus in bread.^48^

At the bioreactor level, ethanol concentrations of 1.8 g/L and 1.3 g/L were observed at 48 h for *A. oryzae* and *R. delemar*, respectively. A decline in ethanol concentration was noted after 72 h, which can be attributed to ethanol consumption by both fungi and ethanol evaporation caused by the reactor’s aeration (Figure 2f).^6^ The disparity in ethanol content between shake flasks and bioreactor cultivation may be influenced by the level of dissolved oxygen, which is higher in bioreactors due to more efficient aeration.^30^ In filamentous fungi, ethanol concentrations above 5% (v/v) can inhibit growth and metabolic activities, while concentrations above 10% (v/v) are typically toxic, leading to significant growth inhibition or cell death.^49^ In this study, especially at the bioreactor level, the ethanol concentration was below 5%, indicating that there was no limitation on the fungal growth.

#### 3.1.2 Morphology of the Recovered Solids

Filamentous fungi create an interconnected network of microfibrillar cells known as mycelia. As fungal microfibers expand, they bind substrate particles together while filling the void spaces.^34^ To study the effect of fungal strain type on the morphology of recovered solids, samples were taken from the solids after harvesting, immediately frozen in liquid nitrogen, and freeze-dried. Subsequently, the samples were imaged using FE-SEM. The images indicate a combination of fungal mycelia and large CP particles. An elongated microfibrillar structure was observed for AO in solids harvested from shake flasks and bioreactors (Figure 3a and b, respectively), whereas RD (Figure 3c and d) exhibited shorter microfibers with broader diameters. According to Svensson et al.,^36^ the mycelium of RD, free from substrate residue (bread waste), exhibited a diameter range of 6–10 μm, whereas the microfibers of AO had diameters ranging from 2 to 6 μm.

**Figure 3:**
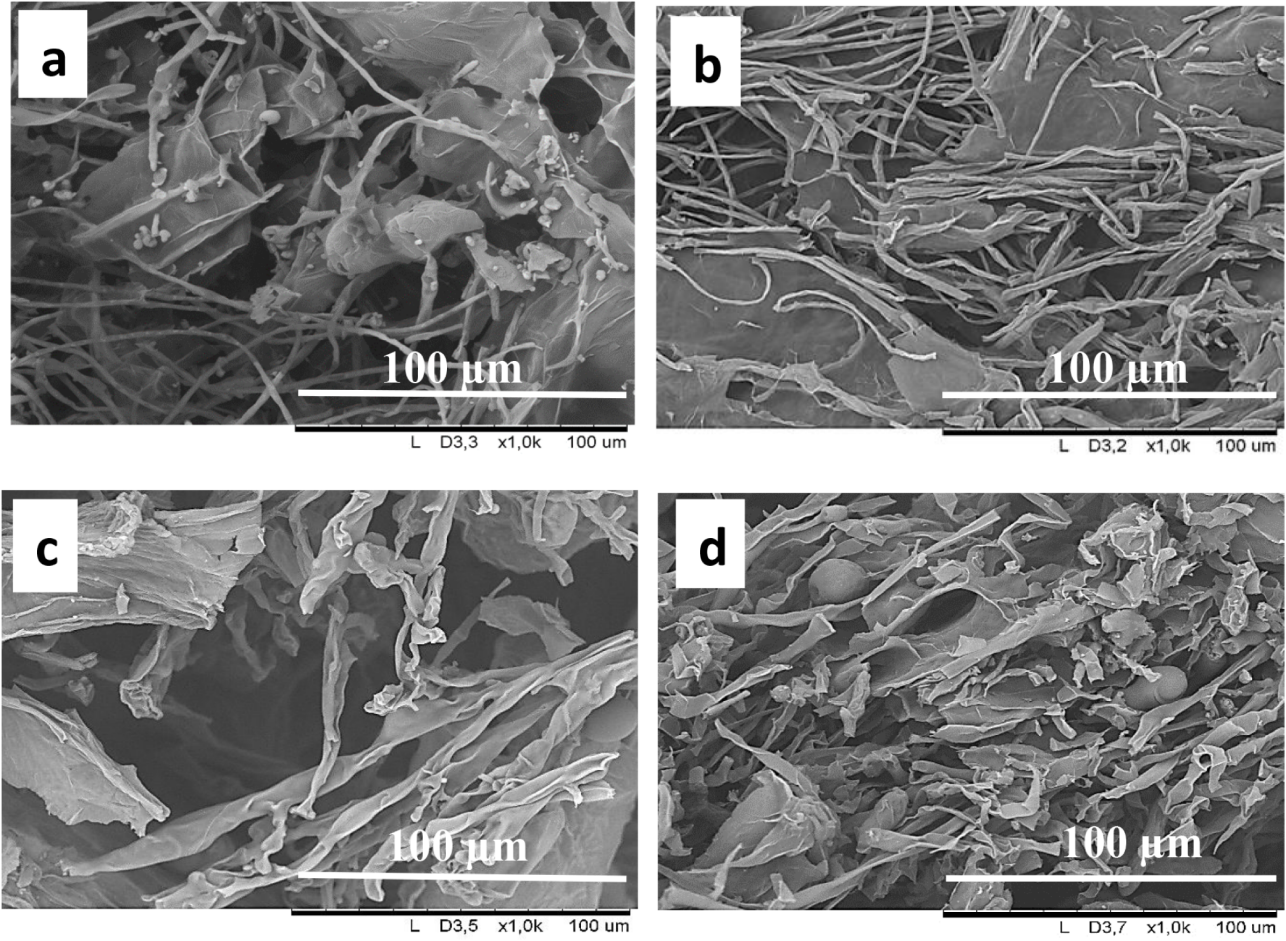
Microscopic images (FE-SEM) of recovered solids after cultivation. Mycelium of AO. between CP particles in shake flask mode (a) and reactors (b), Mycelium of RD between CP particles in shake flask mode (c) and reactors (d).

### 3.2 Mycelium-Based Paper (MBP) Formation and Characterization

#### 3.2.1 Formation of MBP and Its Physical Properties

The recovered solids from the bioreactors were mixed with water to reach a concentration of 2 wt % and then subjected to 5 cycles of grinding using the Supermasscolloider to prepare a homogeneous suspension for the paper preparation. The suspension was further diluted to 0.1% by adding water to facilitate the wet-laid process. The final grammage was set to 120 g/m^2^, which aligns with the commercial packaging paper standard for paper bag applications. Flat sheets were formed using wet-laid suspensions. The filtration time for the production of MBP from the recovered solids suspension was 2.3 and 3.3 min for AO-MBP and RD-MBP, respectively. The percentage of particles that passed through the nylon membrane was 24.1% and 23.3%, respectively. Drying AO-MBP resulted in uniform sheets without any cracks. This study demonstrated the ability of AO-MBP to bend at a 90° angle, without cracking (Figure 4a). In contrast, RD-MBP exhibited brittleness when bent (Figure 4c). The weight, grammage, thickness, and density of AO and RD-MBP were in the same range as those of commercial paper (Table 3). The average pore size of the AO and RD-MBP was recorded at 36.0 and 45.7 μm, respectively. Compared with commercial paper, which exhibits a much denser structure with smaller pore dimensions (2.8 μm), the developed materials show significantly larger average pore sizes. Commercial paper, which is composed of pure cellulose fiber, has a compact structure with low void volume and small pore size.

**Figure 4:**
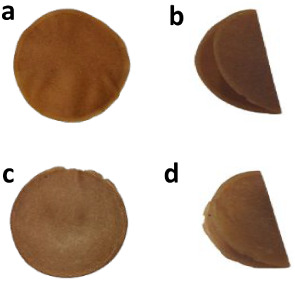
Images of AO-MBP (a), its bending behavior after glycerol-treatment, and (b) RD-MBP (c) and its bending behavior after glycerol-treatment (d).

**Table 3:** Physical Properties of MBP’s are Composed of AO and RD Mycelium

MBP^a^	Weight of MBP with 7 cm diameter (g)	GMS^5^ (g/m^2^)	Thickness (mm)	Density (kg/m^3^)	Average pore size (μm)
Commercial Paper	0.46 ± 0.02	120.8 ± 5.51	0.14 ± 0.00	839.4 ± 5.32	2.8 ± 1.06
AO	0.46 ± 0.01	120.9 ± 4.50	0.13 ± 0.00	911.3 ± 47.2	36.0 ± 0.42
RD	0.48 ± 0.05	123.4 ± 1.82	0.13 ± 0.01	816.7 ± 16.4	45.7 ± 6.08
AO-GLY	0.71 ± 0.07	185.8 ± 1.83	0.16 ± 0.05	1135.9 ± 48.0	29.3 ± 1.62
RD-GLY	0.71 ± 0.02	184.5 ± 7.35	0.21 ± 0.01	870.19 ± 37.7	29.8 ± 1.9
AO-PRESS	0.47 ± 0.00	123.4 ± 1.83	0.10 ± 0.00	1136.4 ± 35.3	21.5 ± 1.83
RD-PRESS	0.46 ± 0.01	119.5 ± 4.50	0.10 ± 0.00	1166.5 ± 23.7	20.0 ± 0.72

a GLY refers to glycerol and PRESS refers to hot-press treatment.

To enhance flexibility, treatment with glycerol as a plasticizer was examined. Following glycerol treatment, both AO-MBP and RD-MBP were completely folded (Figure 4b and d). The weight of the MBP increased by 54% and 48% for the AO-MBP and RD-MBP, respectively, after treatment, demonstrating the penetration of glycerol into the MBP structure. Moreover, the grammage, thickness, and density increased, while the average pore size decreased, indicating a pore-blocking effect of glycerol on both AO-MBP and RD-MBP. Appeal et al.^50^ similarly reported a reduction in air voids in mycelium films treated with 8% glycerol compared to untreated samples. The effects of glycerol on polymer systems are complex and can vary depending on the specific polymer composition and structure. The uptake of glycerol into the porous structure of the mycelium sheet may influence on pore size while glycerol interacts with biopolymer available in mycelium as well as carrot residue primarily through hydrogen bonding.^30^ Owing to its multiple hydroxyl groups, glycerol forms hydrogen bonds with polysaccharide and protein chains within the matrix, thereby disrupting interchain hydrogen bonding and reducing intermolecular interactions. This increases polymer chain mobility and enhances flexibility.^50^ Similar behavior has been reported in cellulose-based systems^51^ and is likewise relevant for mycelium-based materials, where glycerol acts through two primary mechanisms: (i) increasing intermolecular spacing within the polysaccharide-rich network (e.g., chitin and glucans), thereby reducing structural packing density, and (ii) modifying hydrogen-bonding interactions within and between polymer chains; together, these effects enhance chain mobility and overall flexibility. However, it is important to note that such interactions may depend on the specific polysaccharide components present, as highly ordered structures such as cellulose may be less susceptible to disruption of intrachain hydrogen bonding compared to more amorphous polysaccharides such as hemicelluloses.^52^ Additionally, the glycerol–water solution used for post-treatment induces swelling in the mycelium matrix, further contributing to its flexibility.^53^ Shao et al.^18^ similarly reported hydrogen bonding between glycerol and polysaccharides and proteins in mycelium-based films, leading to modified mechanical properties. A comparable plasticization mechanism likely explains the changes observed in the MBP samples in this study. Applying a hot-press to the untreated MBP reduced the thickness from 0.13 mm to 0.10 mm for both MBPs, resulting in an MBP with higher density (1136.4 and 1166.5 kg/m^3^ for AO-MBP and RD-MBP, respectively). Similarly, hot-press treatment has been reported to enhance the density of bulk mycelium-based composites.^54^ The apparent average pore size decreased to approximately 20 μm after the hot-press treatment. Despite improved densification, the resulting MBP exhibited extremely brittle behavior when folded, restricting its application as a packaging material.

#### 3.2.2 Scanning Electron Microscopy (SEM) of MBP

FE-SEM images were captured from the surface and cross-section of MBP to analyze its surface morphology and microstructure (Figure 5). The morphology of MBP was compared with that of commercial paper produced from virgin fiber through industrial papermaking for paper bags available in the market. This comparison was aimed at evaluating the efficiency of MBP for this specific application. Figure 5a shows a surface image of commercial paper, presenting a high-density structure of intertwined cellulose fibers of different shapes and sizes. The cross-sectional image of this paper (Figure 5b) shows the cellulose fibers arranged in compacted layers.

**Figure 5:**
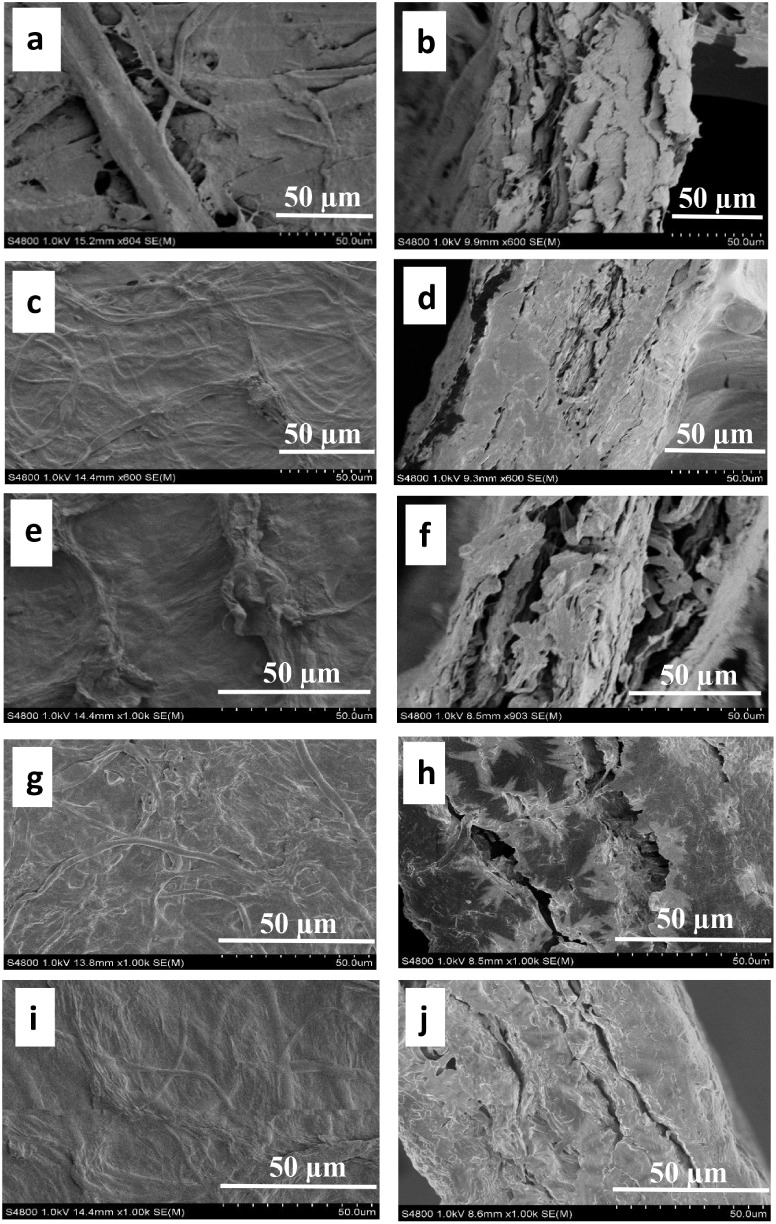
Microscopic images (FE-SEM) of the surfaces (a, c, e, g and (i) and cross sections of different papers (b, d, f, and h). (a) and (b): Commercial paper, (c) &; (d): AO-MBP, (e) and (f): RD-MBP, (g) and (h): AO-MBP (Glycerol), and (i) and (j): AO-MBP (Pressed).

The SEM image of the surface of AO-MBP (Figure 5c) exhibited a densely bound surface morphology, where the mycelium with a long microfibrillar structure was distributed in the matrix of CP particles. The cross-sectional image of the same specimen (Figure 5d) shows a closely packed parallel structure. The surface (Figure 5e) and cross-section (Figure 5f) of RD-MBP showed a less organized structure compared to the AO-MBP. The microfibrillar structure was not clearly visible, as shown in the AO-MBP images. This might be due to the microfibrillar structure of RD, which had a larger diameter and shorter length than AO, that merged with the structure of the CP matrix during the wet-laid process and drying. The loosely packed structure of RD-MBP, as depicted in the cross-sectional image (Figure 5f), may also be related to the shorter microfibers in RD compared to AO, resulting in a lower ability to bind the CP particles and form an interconnected compact structure. Shao et al.^18^ noted that the mycelium (*Auricularia polytricha*) has a loose tubular structure, which reflects the poor mechanical properties of the film. Similar images of the surface of a mycelium-based composite with different substrates were observed by Peng et al.^55^

Following glycerol treatment, the SEM image showed a smoother surface for AO-MBP, where the microfibrillar structure was visible (Figure 5g). The cross-sectional image of this paper (Figure 5h) shows a swollen structure compared to the respective image for the untreated paper (Figure 5b). This may be due to the diffusion of glycerol into the structure of the paper during pretreatment. Moreover, under the hot compression treatment of AO-MBP, the surface of MBP became flattened (Figure 5i), and the pores were significantly reduced in the cross-section image (Figure 5j).

#### 3.2.3 Color of MBP

Color detection by the naked eye can often be inaccurate; hence, color parameters were determined for this purpose. CIE Lab color parameters were measured, where L* represents how light or dark the color appears. Higher values indicate lighter colors, whereas lower values represent darker colors. The parameter a* measures the degree of greenness (−) or redness (+) of the colors. Negative values indicate greener colors, while positive values indicate redder colors. Similarly, b* quantifies the amount of blueness (−) or yellowness (+) in the color, with negative values indicating bluer tones and positive values indicating yellower tones. This model is illustrated in Figure S6 in the supplementary file. The colors of the MBPs were determined using colorimetry, and the corresponding results for the color parameters are listed in Table 4. Commercial paper exhibits lighter colors (L*: 62.2) compared to the MBP. The L* value increased after adding glycerol from 44.7 to 49.5 for AO-MBP and from 49.3 to 52.6 for RD-MBP, resulting in lighter color. These characteristics are particularly desirable in packaging materials, as they enhance the visual appeal and consumer perception of the product, aligning with the preference for light and neutral-toned packaging in the markets. Applying hot-press led to a decrease in the L* value to 41.5 and 45.4 with in for AO and RD-MBP, respectively. Moreover, for different MBPs, b*, which signifies the presence of yellow color, and a*, which represents the red color, were not significantly different from commercial paper, regardless of the type of fungus and the pretreatment conditions.

**Table 4:** Color Parameters (L*, a^*^, and b^*^) of Industrial Paper and MBP

**Type of MBP**	L*	a*	b*
Commercial Paper	62.2 ± 0.43	7.6 ± 0.62	21.5 ± 0.48
AO	44.7 ± 0.85	8.6 ± 1.03	21.7 ± 0.80
RD	49.3 ± 1.23	7.2 ± 1.18	21.8 ± 1.29
AO-GLY	49.5 ± 0.73	6.9 ± 0.86	20.5 ± 0.83
RD- GLY	52.6 ± 0.89	7.3 ± 1.22	23.1 ± 1.05
AO-PRESS	41.5 ± 0.65	7.9 ± 1.28	19.4 ± 1.10
RD-PRESS	45.4 ± 0.78	8.3 ± 1.06	21.3 ± 0.98

#### 3.2.4 Mechanical Properties of MBP

The mechanical properties of mycelium-based materials vary depending on the fungal strain, substrate, and growth conditions.^56^ Analysis of the mechanical properties of MBP, including the tensile strength, elongation at break, and E modulus (Table 5), revealed significant differences among the MBPs prepared in this study (*p* < 0.05). The AO-MBP exhibited a high tensile strength of 48.8 MPa, which is approximately 72% of the tensile strength of commercial paper made from high-quality cellulose pulp. This may be attributed to the long microfibrillar structure of the AO mycelium, which not only acted as a binder for the CP particles but also significantly reinforced the formed matrix. In contrast, RD-MBP displayed a lower tensile strength (31.5 MPa) due to the shorter microfibers of this fungus. RD-MBP also showed higher average pore size, as indicated in Table 2, which indicates a limitation in forming a strong bond between the mycelium and the CP particles. Notably, these findings align with the tensile (15–55 MPa) and elongation at break (2–8%) reported for chitosan films by Mahapatra et al.^57^

**Table 5:** Mechanical Properties of MBP

Type of MBP	Tensile strength (MPa)	Elongation at break (%)	E modulus (MPa)
Commercial paper	67.3 ± 2.45	7.7 ± 0.35	2032.3 ± 24
AO	48.8 ± 6.05	2.4 ± 0.1	3251.1 ± 26
RD	31.5 ± 2.10	2.9 ± 1.5	2737.3 ± 47
AO-GLY	9.2 ± 0.55	6.8 ± 1.6	754.8 ± 99
RD-GLY	8.5 ± 1.25	7.3 ± 1.8	586.9 ± 11
AO-PRESS	61.8 ± 5.25	4.8 ± 0.32	2709.2 ± 12
RD-PRESS	43.8 ± 2.40	4.4 ± 0.85	2244.5 ± 18

Upon treating the MBP with glycerol, the tensile strength was reduced to 9.23 and 8.56 MPa for the AO and RD-MBP, respectively. However, it led to an increase in elongation at break from 2.4 to 6.8% for the AO-MBP and from 2.9 to 7.3% for the RD-MBP. This significantly enhances the flexibility of MBP, making it suitable for applications such as wrapping paper. Glycerol acts as a plasticizer, due to its hygroscopic nature, enhancing the molecular chain mobility by filling the gaps between polymer chains, thereby increasing the flexibility of biomaterials.^18,50^ Similarly, the ductility of chitosan films was improved by glycerol treatment.^58^

Furthermore, subjecting AO and RD-MBP to hot-press resulted in a significant increase in tensile strength, reaching 61.88 and 43.84 MPa, respectively, as shown in Table 5. Hot-pressing contributes to the reduction of material average pore size by collapsing and welding internal pores or defects. This process enhances fiber bonding, allowing fibers to bond more effectively and creating stronger interfiber connections, which in turn improves tensile strength.^59^ These values represent 92% and 65% of the tensile strength of the commercial paper, respectively. Additionally, the elongation at break and E modulus exhibited higher values after the post-treatment. Despite their promising mechanical properties, the obtained papers exhibited brittle behavior, which restricted their applications.

Ashby’s bubble chart was employed to determine the position of mycelium-based papers compared to commercially available materials and to evaluate their potential for different applications. This chart serves as a graphical and visual tool, dividing different materials into regions representing various classes or families of materials, such as plastics, natural fibers, metals, wood and paper materials, and composites, based on their properties. In Figure 6, each commercial material is represented by a bubble in the plot. The small red points on the chart correspond to the MBPs developed in this study, and the orange points are attributed to commercial paper. Accordingly, our MBP were positioned within bubbles of wood and paper materials.

**Figure 6:**
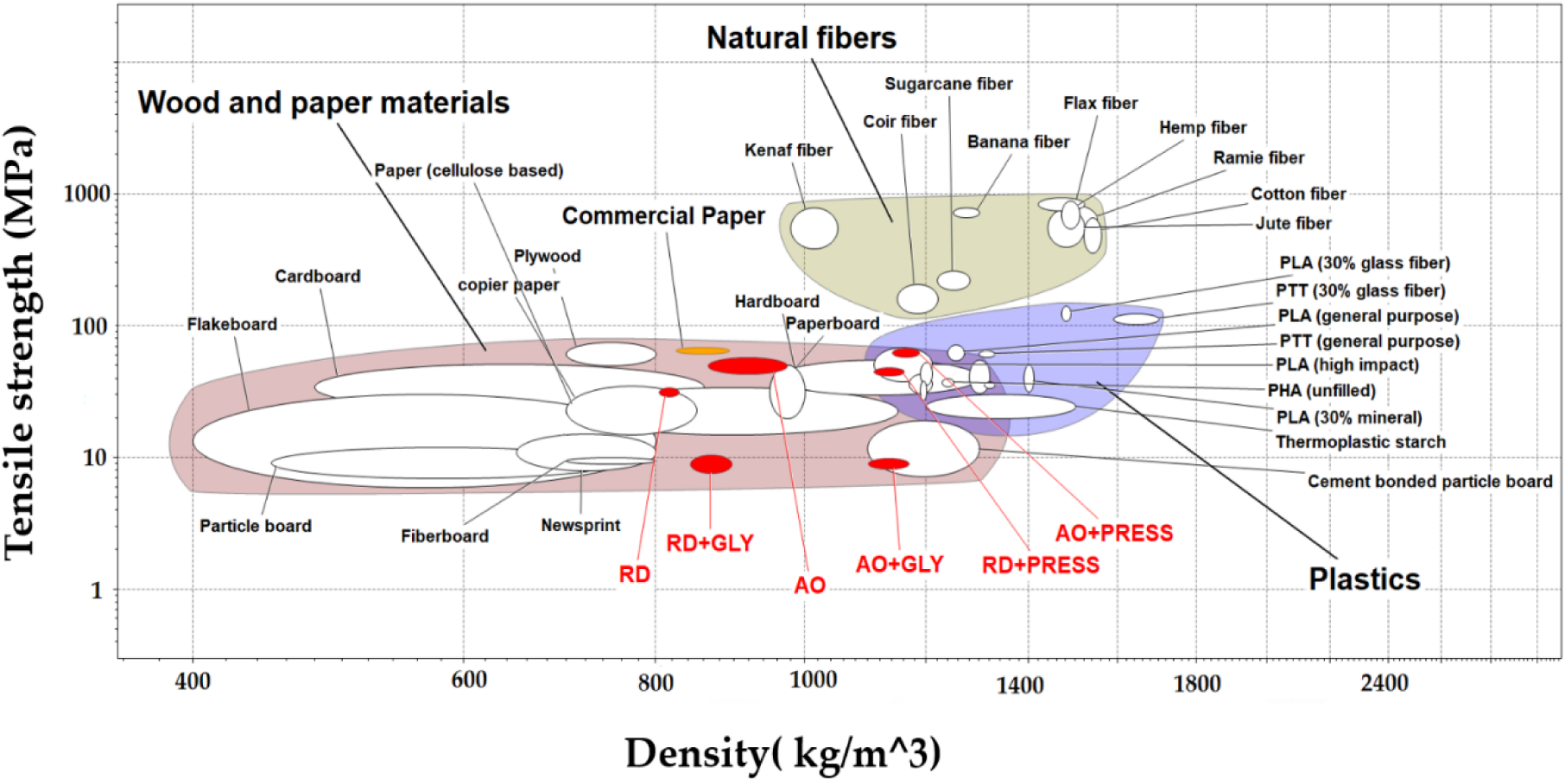
Ashby chart of tensile strength versus density for material classification (chart reproduced from CES Edu Pack 2021, ANSYS Granta 2021 Granta Design). The products developed in this study are positioned relative to commercially established materials for performance benchmarking. The materials evaluated include RD, AO, RD+GLY, AO+GLY, RD+PRESS, and AO+PRESS.

#### 3.2.5 Thermal Analysis of MBP

Thermogravimetric analysis (TGA) was conducted to study the thermal degradation of the MBPs. The thermal degradation behavior of the MBPs under a constant temperature increase is presented in Figure 7. The thermal behavior of AO-MBP and commercial paper exhibited a single stage of degradation, attributed to the presence of biopolymers such as polysaccharides, lipids, and proteins. The initial mass loss (approximately 50–120 °C) is attributed to the evaporation of water and volatile extractives. Subsequent degradation between 250 and 380 °C is associated with the breakdown of organic substances such as polysaccharides and proteins found in the mycelium and cellulosic fraction of CP.^15,23^ Meanwhile, MBP treated with glycerol (AO-GLY-MBP) displayed a distinct degradation behavior between 180 and 220 °C, which was linked to glycerol, as supported by derivative thermogravimetric analysis (DTGA) curves (Figure 7b). The temperature range 380 and 600 °C indicates the decomposition of the remaining char, resulting in a residual mass (char residue) ranging from 20% to 33%.^60^ Shao et al.^18^ observed a second-stage decomposition in the thermal degradation of films from mushroom mycelium, attributed to glycerol evaporation occurring between 180 and 280 °C. Additionally, they noted a third degradation phase starting around 270 °C and finishing close to 400 °C, suggesting an interaction between glycerol and mycelium. This interaction may contribute to the plasticizing effect of glycerol on the mycelium films. Similar patterns were observed for RD-MBP and its glycerol-treated.

**Figure 7:**
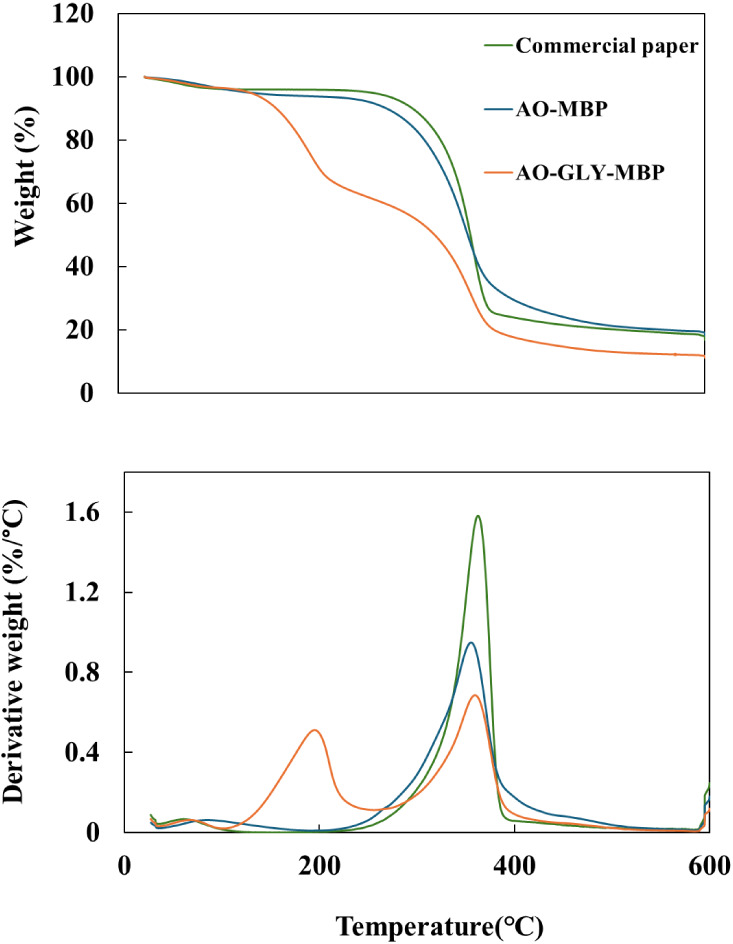
(a) Thermogravimetric analysis and (b) derivative thermogravimetric analysis of commercial paper and MBP.

#### 3.2.6 Differential Scanning Calorimetry (DSC)

Differential scanning calorimetry (DSC) was used to study the thermal transitions of the MBP. The DSC graphs in Figure 8 show endothermic transitions at approximately 90 °C, which corresponds to the glass transition temperature (*T*_*g*_) of chitin in the mycelium.^61^ Local relaxation of the backbone chain of chitin causes this transition.^62^ A similar behavior is observed for commercial paper, which is likely related to moisture effects or minor additives rather in post treatment of commercial paper than a true glass transition of cellulose. An endothermic transition was observed at around 270 °C for the post-treated samples, which is due to the presence of glycerol. An exothermic transition was noticed at around 350 °C. While chitin is thermally stable due to hydrogen bonding, this transition can be attributed to the decomposition of the acetyl-glucosamine unit of chitin and evaporation of volatile low molecular products during depolymerization.^63,64^ The effect of glycerol can be noticed in both endothermic and exothermic curves.

**Figure 8:**
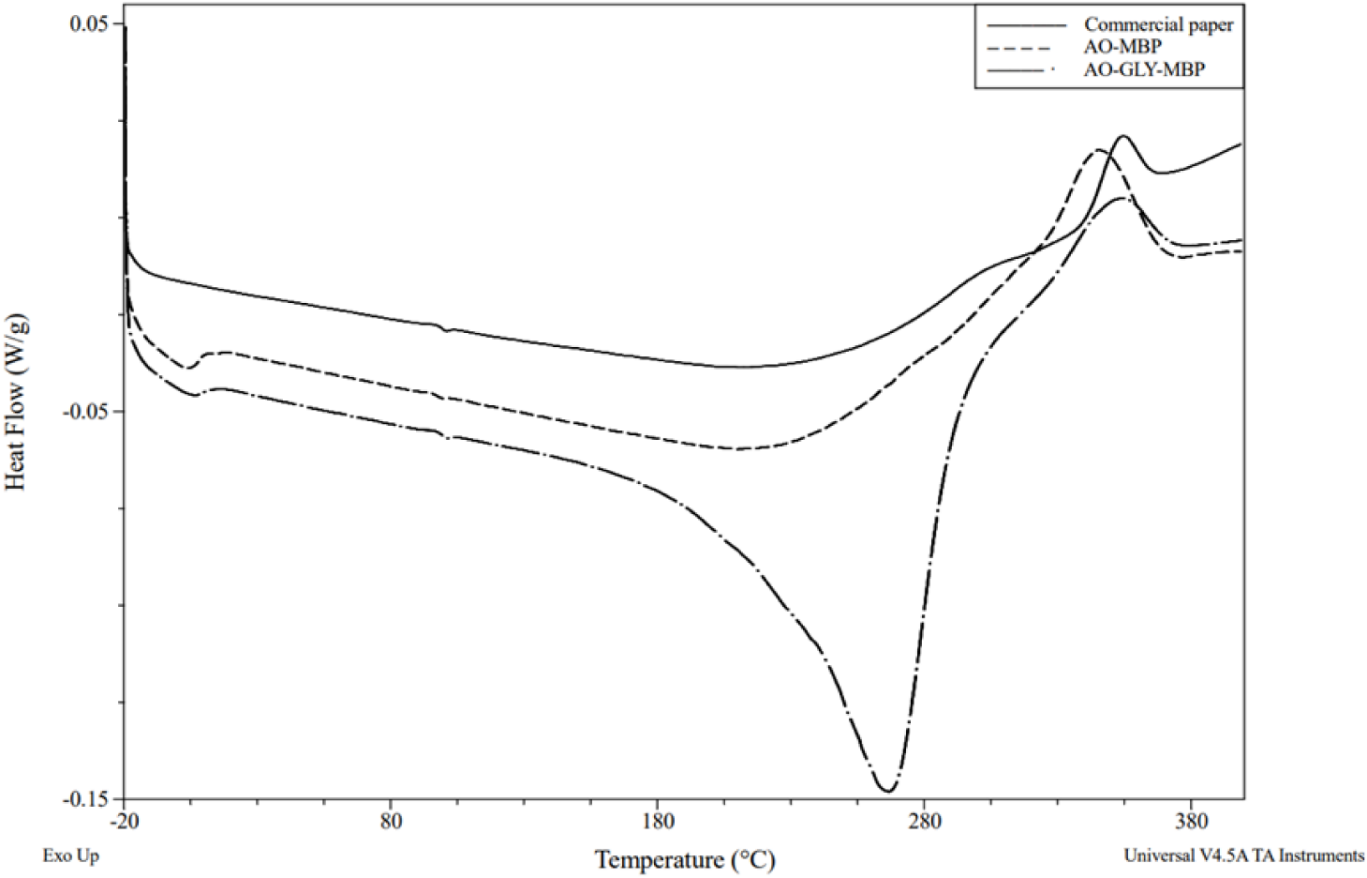
DSC curve of commercial packaging paper and MBP.

#### 3.2.7 Fourier Transform Infrared (FT-IR) Spectra

FTIR analysis was employed to investigate the chemical composition of MBP and the effect of glycerol on the chemical structure. Figure 9 present the peak at 3300 cm^–1^, which was attributed to the stretching vibration of the O–H groups in cellulose and hemicellulose, which are available in commercial paper and CP.^55^ In AO-GLY-MBP, glycerol treatment caused broading stretching vibration peak of the hydroxyl groups exhibited broadening, hinting at the potential interaction between glycerol and polysaccharides in the *A. oryzae* hyphae and cellulosic component of the MBP. A distinctive band of the fungal mycelium appeared at 2920 cm^–1^, which was attributed to the stretching vibration of the C–H groups in fatty acids and lipids, this peak was not seen in commercial paper.^21^ The stretching vibration of the carbonyl in acetyl group of hemicelluloses and methyl ester in pectin was noted at 1730 cm^–1^. The stretching vibration of the C = C or N–H groups at 1630 cm^–1^ is related to amide I within the fungal mycelium. Additionally, bands at 1241 and 1548 cm^–1^ correspond to the stretching vibration of N–H and C–N groups in amide II and amide III, respectively, in the fungal mycelium, with the band at 1326 cm^–1^ representing the stretching vibration of NH_2_ in the mycelial protein.^23^ The peak at 1241 cm^–1^ in the amide III region, associated with C–N stretching and N–H bending, decreased to 1237 cm^–1^ in the glycerol-treated samples, potentially due to the reduction of surface hydrophobins when submerged in a glycerol bath.^65^

**Figure 9:**
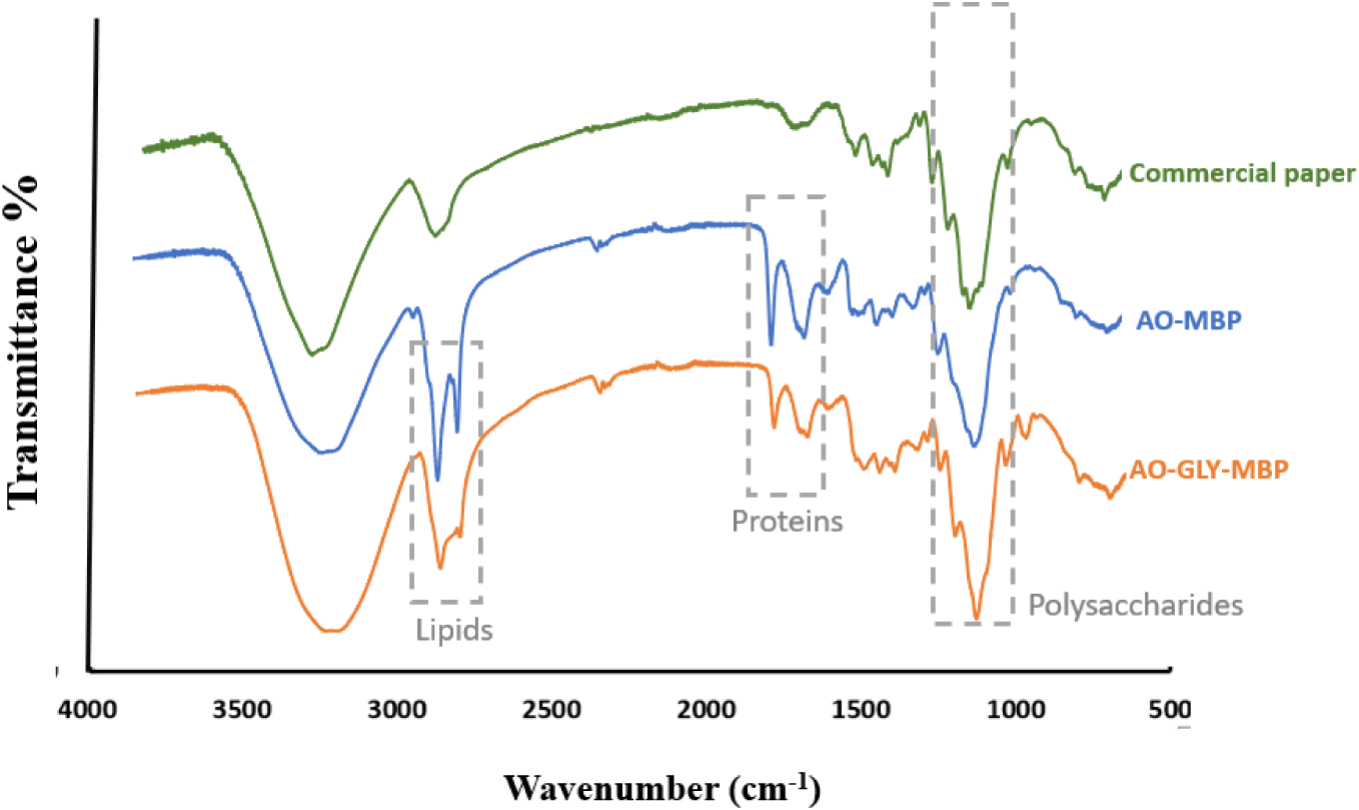
Fourier transforms infrared spectroscopy of commercial paper and MBP.

#### 3.2.8 Contact Angle Analysis

Contact angle analysis was performed to evaluate the surface hydrophobicity of the papers, which is critical for assessing moisture resistance in paper packaging materials. Higher contact angle values indicate increased material hydrophobicity, while lower values suggest greater hydrophilicity. As illustrated in Figure 10, commercial paper exhibits superior hydrophobic behavior with a contact angle of 95°. MBP derived from *Aspergillus Oryza* mycelium, displayed a higher contact angle (57°) compared to the RD-MBP (23°). Statistical analysis indicated significant differences among the MBPs (*P* < 0.05), except between RD-MBP and RD-GLY- MBP. The limited water affinity of the AO-MBP may be linked to the presence of hydrophobic components in the outer layer of the fungal cell wall.^18^ The lower contact angle of the RD-MBP may be attributed to the higher porous structure and surface topography of this MBP. However, the contact angle decreased to 34° and 21° for the AO and RD-MBP, respectively, after glycerol treatment. This aligns with the findings of Shao et al.,^18^ who reported that glycerol treatment leads to a decreased contact angle of less than 40° in pure mycelium films due to interaction with the mycelium, imparting hydrophilic properties to the mycelium-based films. Typically, the distinct wettability between fungal species necessitates further research to identify suitable species with enhanced hydrophobicity. Additionally, the substrate composition can affect the contact angle of mycelium-based material.^18^ Appel et al.^50^ reduced the contact angle from 120° in an untreated sample to 49° in a mycelium film by submerging it in a 32% aqueous glycerol solution for 24 h. Similarly, cellulose-chitosan films derived from mango leaves with the addition of 1% glycerol exhibited a contact angle of 69°.^66^ Moreover, the presence of hydrophobin proteins and lipids in filamentous fungi can significantly increase the hydrophobicity of mycelium based materials.^67^ As demonstrated by Sun et al.,^65^ the removal of hydrophobins from the mycelium using formic acid results in the loss of hydrophobic ability.

**Figure 10:**
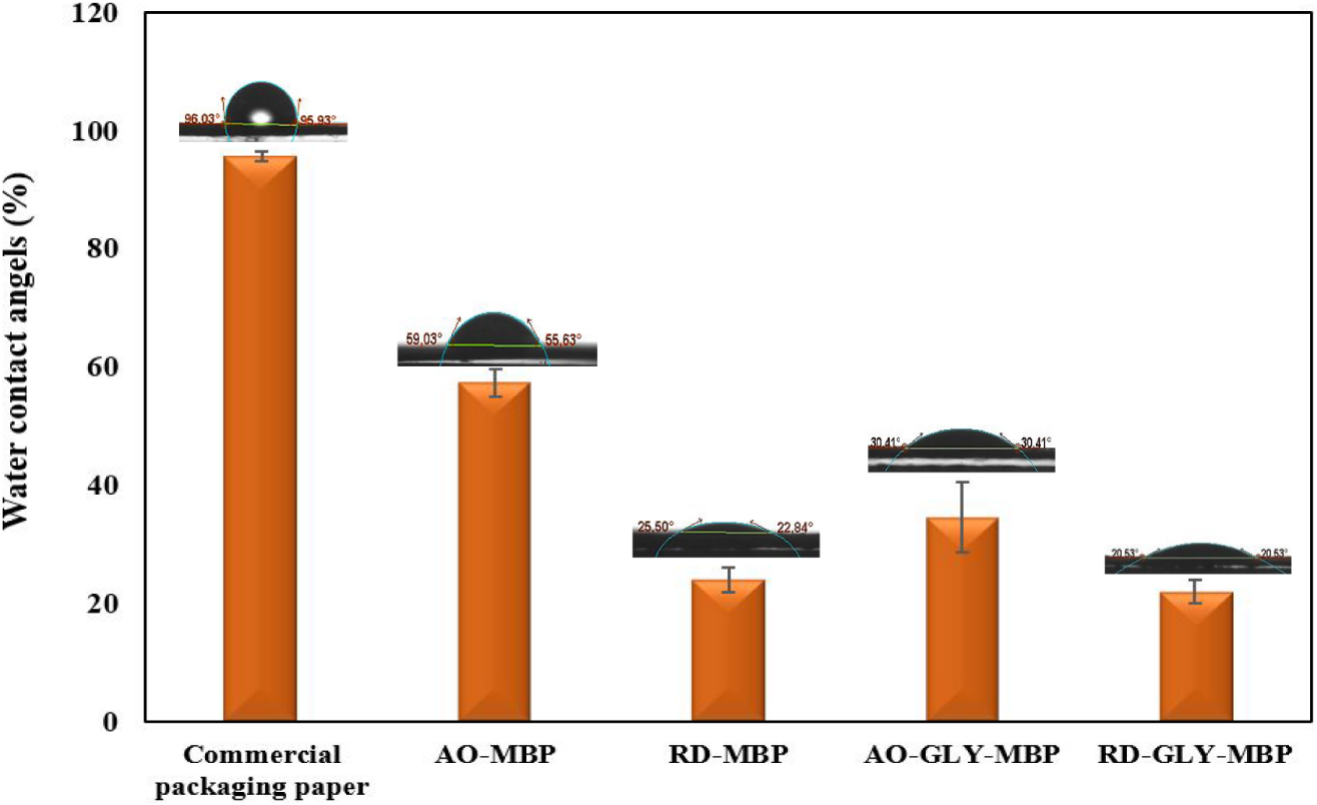
Water contact angle of commercial paper and different MBP developed in the study.

## 4 Conclusion

Mycelium-based paper (MBP) was successfully fabricated from solid material harvested after submerged cultivation of *A. oryzae* and *R. delemar* on whole CP. The harvested material containing mycelium and residues of CP particles was subjected to a wet-laid process to prepare MBPs. By using the entire pomace without prior extraction or fractionation, the approach aligns with circular-material principles and reduces processing complexity. In this product, the mycelium acted as an adhesive, binding the carrot pomace particles together and eliminating the need for oil-based binder. The distinct mycelial morphologies of two strain fungi demonstrated a clear difference in MBP structure and performance, producing a denser and stronger paper than *R. delemar*, with a tensile strength of 49 MPa and pore sizes similar to those of papers made from refined cellulose fibers. Post-treatments further broadened the material’s property range. Glycerol impregnation significantly improved flexibility of MBP, raising elongation at break by 60%. In contrast, hot-pressing enhanced density and tensile strength, though at the expense of flexibility. Ashby plots confirmed that the MBPs occupy the material space of wood- and paper-based products, demonstrating their competitiveness with existing cellulosic packaging materials.

This study provides a foundation for the development of mycelium-based paper from abundant food waste, achieving properties comparable to commercial paper. The combination of tunable mechanics, biodegradability, and reliance on food-industry side streams underscores its potential for sustainable packaging. Future work should target improved hydrophobicity, enhanced barrier performance, and assessment of process scalability for industrial adoption.

## References

[1] Berglund L., Noël M., Aitomäki Y., Öman T., Oksman K. (2016). Production potential of cellulose nanofibers from industrial
residues: Efficiency and nanofiber characteristics. Ind. Crops Prod.

[2] Chojnacki J., Zdanowicz A., Ondruška J., Šooš L'., Smuga-Kogut M. (2021). The Influence
of Apple, Carrot and Red Beet Pomace
Content on the Properties of Pellet from Barley Straw. Energies.

[3] Szymańska-Chargot M., Chylińska M., Gdula K., Kozioł A., Zdunek A. (2017). Isolation and characterization of cellulose from different
fruit and vegetable pomaces. Polymers.

[4] Nawirska A., Kwaśniewska M. (2005). Dietary fibre
fractions from fruit and vegetable processing
waste. Food Chem.

[5] Sharma K. D., Karki S., Thakur N. S., Attri S. (2012). Chemical composition,
functional properties and processing of carrot—a review. J. Food Sci. Technol.

[6] Rajinipriya M., Nagalakshmaiah M., Robert M., Elkoun S. (2018). Homogenous and transparent
nanocellulosic films from carrot. Ind. Crops
Prod.

[7] Szymańska-Chargot M., Chylińska M., Pertile G., Pieczywek P. M., Cieślak K. J., Zdunek A., Frąc M. (2019). Cellulose.

[8] Sogut E., Cakmak H. (2020). Utilization of carrot
(Daucus carota L.) fiber as a
filler for chitosan based films. Food Hydrocolloids.

[9] Yoon K. Y., Cha M., Shin S. R., Kim K. S. (2005). Enzymatic production of a soluble-fibre
hydrolyzate from carrot pomace and its sugar composition. Food Chem.

[10] Trabert A., Schmid V., Keller J., Emin M. A., Bunzel M. (2022). Chemical composition
and technofunctional properties of carrot (Daucus carota L.) pomace
and potato (Solanum tuberosum L.) pulp as affected by thermomechanical
treatment. Eur. Food Res. Technol.

[11] De
Vrije T., Budde M. A., Lips S. J., Bakker R. R., Mars A. E., Claassen P. A. (2010). Hydrogen production from carrot pulp
by the extreme thermophiles Caldicellulosiruptor saccharolyticus and
Thermotoga neapolitana. Int. J. Hydrogen Energy.

[12] Khoshkho S. M., Mahdavian M., Karimi F., Karimi-Maleh H., Razaghi P. (2022). Production of bioethanol from carrot pulp in the presence
of Saccharomyces cerevisiae and beet molasses inoculum; a biomass
based investigation. Chemosphere.

[13] Zhao L., Ma Y., Awasthi M. K., Tian D., He J., Huang M., Zou J., Lei Y., Shen F. (2025). Enhancement of bacterial cellulose
production synergistic H2 and volatile fatty acids from fruit and
vegetable waste through retting pretreatment. Ind. Crops Prod.

[14] Makharita R. R., El-Ahmady El-Naggar N., Baghdadi A. M., Hamouda R. A. (2025). Sustainable production
of bacterial nanocellulose from date fruit waste using Bacillus haynesii
for waste valorisation and crystal violet dye removal. Sci. Rep.

[15] Mousavi S. N., Parchami M., Ramamoorthy S. K., Soufiani A. M., Hakkarainen M., Zamani A. (2023). Bioconversion of Carrot
Pomace to Value-Added Products:
Rhizopus delemar Fungal Biomass and Cellulose. Fermentation.

[16] Yang L., Park D., Qin Z. (2021). Material function of mycelium-based
bio-composite: A review. Front. Mater.

[17] Chandra D. K., Kumar A., Mahapatra C. (2024). Ecofriendly
bioplastics from biowaste:
Antimicrobial and functional enhancements for sustainable packaging. Eur. Polym. J.

[18] Shao G., Xu D., Xu Z., Jin Y., Wu F., Yang N., Xu X. (2024). Green and sustainable
biomaterials: Edible Bioplastic films from
mushroom mycelium. Food Hydrocolloids.

[19] Verma N., Jujjavarapu S. E., Mahapatra C. (2023). Green sustainable biocomposites:
Substitute to plastics with innovative fungal mycelium based biomaterial. J. Environ. Chem. Eng.

[20] Lingam D., Narayan S., Mamun K., Charan D. (2023). Engineered
mycelium-based
composite materials: Comprehensive study of various properties and
applications. Constr. Build. Mater.

[21] Joshi K., Meher M. K., Poluri K. M. (2020). Fabrication
and Characterization
of Bioblocks from Agricultural Waste Using Fungal Mycelium for Renewable
and Sustainable Applications. ACS Appl. Bio
Mater..

[22] Rathinamoorthy R., Bharathi T. S., Snehaa M., Swetha C. (2023). Structural and Chemical
Characterization of Mycelium Sheets Developed from Penicillium Camemberti. J. Polym. Environ.

[23] Haneef M., Ceseracciu L., Canale C., Bayer I. S., Heredia-Guerrero J. A., Athanassiou A. (2017). Advanced materials from fungal mycelium: fabrication
and tuning of physical properties. Sci. Rep.

[24] Antinori M. E., Contardi M., Suarato G., Armirotti A., Bertorelli R., Mancini G., Debellis D., Athanassiou A. (2021). Advanced mycelium
materials as potential self-growing biomedical scaffolds. Sci. Rep.

[25] Heide A., Wiebe P., Sabantina L., Ehrmann A. (2023). Suitability of Mycelium-Reinforced
Nanofiber Mats for Filtration of Different Dyes. Polymers.

[26] Cesar E., Canche-Escamilla G., Montoya L., Ramos A., Duarte-Aranda S., Bandala V. M. (2021). Characterization and physical properties of mycelium
films obtained from wild fungi: natural materials for potential biotechnological
applications. J. Polym. Environ.

[27] Sanchez-Díaz M. R., Lazarte M. S., Moavro A., Peltzer M. A., Ludemann V. (2023). Naturally
Multicomponent Materials Obtained from Filamentous Fungi: Impact of
Different Cell Rupture Treatment on Film Properties. J. Polym. Environ.

[28] French V., Du C., Foster E. J. (2023). Mycelium
as a self-growing biobased material for the
fabrication of single-layer masks. J. Bioresour.
Bioprod.

[29] Köhnlein M. B. M., Abitbol T., Oliveira A. O., Magnusson M. S., Adolfsson K. H., Svensson S. E., Ferreira J. A., Hakkarainen M., Zamani A. (2022). Bioconversion of food waste to biocompatible wet-laid
fungal films. Mater. Des.

[30] Wijayarathna E. R. K. B., Mohammadkhani G., Soufiani A. M., Adolfsson K. H., Ferreira J. A., Hakkarainen M., Berglund L., Heinmaa I., Root A., Zamani A. (2022). Fungal textile alternatives from
bread waste with leather-like properties. Resour.,
Conserv. Recycl.

[31] Svensson S. E., Ferreira J. A., Hakkarainen M., Adolfsson K. H., Zamani A. (2021). Fungal textiles: Wet spinning of fungal microfibers
to produce monofilament yarns. Sustainable Mater.
Technol..

[32] Attias N., Reid M., Mijowska S. C., Dobryden I., Isaksson M., Pokroy B., Grobman Y. J., Abitbol T. (2021). Biofabrication of nanocellulose–mycelium
hybrid materials. Adv. Sustainable Syst..

[33] Irbe I., Filipova I., Skute M., Zajakina A., Spunde K., Juhna T. (2021). Characterization of
novel biopolymer blend mycocel from plant cellulose
and fungal fibers. Polymers.

[34] Vandelook S., Elsacker E., Van Wylick A., De Laet L., Peeters E. (2021). Current state
and future prospects of pure mycelium materials. Fungal. Biol. Biotechnol.

[35] Mousavi S. N., Ramamoorthy S. K., Hakkarainen M., Zamani A. (2024). Production of Mycelium-Based
Papers from Carrot Pomace and Their Potential Applications for Dye
Removal. J. Polym. Environ.

[36] Svensson, S. Development of Filaments Using Cell Wall Material of Filamentous Fungi Grown on Bread Waste for Application in Medical Textiles. Högskolan i Borås. 2024.

[37] Braho V., Sar T., Taherzadeh M. J. (2024). Cultivation of edible filamentous
fungi on pomegranate by-products as feedstocks to produce mycoprotein. Syst. Microbiol. Biomanuf.

[38] Vendruscolo F., Albuquerque P. M., Streit F., Esposito E., Ninow J. L. (2008). Apple pomace:
a versatile substrate for biotechnological applications. Crit. Rev. Biotechnol.

[39] Vieira M. G. A., da Silva M. A., dos Santos L. O., Beppu M. M. (2011). Natural-based plasticizers
and biopolymer films: A review. Eur. Polym.
J.

[40] Sar T., Ferreira J. A., Taherzadeh M. J. (2021). Conversion
of fish processing wastewater
into fish feed ingredients through submerged cultivation of Aspergillus
oryzae. Syst. Microbiol. Biomanuf.

[41] Daba G. M., Mostafa F. A., Elkhateeb W. A. (2021). The ancient
koji mold (Aspergillus
oryzae) as a modern biotechnological tool. Bioresour.
Bioprocess..

[42] Miyazawa K., Yoshimi A., Sano M., Tabata F., Sugahara A., Kasahara S., Koizumi A., Yano S., Nakajima T., Abe K. (2019). Both galactosaminogalactan and α-1, 3-glucan contribute to
aggregation of Aspergillus oryzae hyphae in liquid culture. Front Microbiol.

[43] Ullmann C., Babick F., Stintz M. (2019). Microfiltration
of submicron-sized
and nano-sized suspensions for particle size determination by dynamic
light scattering. Nanomaterials.

[44] Kawa-Rygielska J., Pietrzak W., Lennartsson P. R. (2022). High-efficiency
conversion of bread
residues to ethanol and edible biomass using filamentous fungi at
high solids loading: A biorefinery approach. Appl. Sci.

[45] Souza
Filho P. F., Zamani A., Taherzadeh M. J. (2019). Edible
protein production by filamentous fungi using starch plant wastewater. Waste Biomass Valorization.

[46] Adnan M., Zheng W., Islam W., Arif M., Abubakar Y. S., Wang Z., Lu G. (2018). Carbon catabolite
repression in filamentous
Fungi. Int. J. Mol. Sci.

[47] Satari B., Karimi K., Taherzadeh M. J., Zamani A. (2016). Co-production of fungal
biomass derived constituents and ethanol from citrus wastes free sugars
without auxiliary nutrients in airlift bioreactor. Int. J. Mol. Sci.

[48] Wijayarathna, E. R. K. B. Development of Fungal Leather-like Material from Bread Waste, Master’s Thesis University of Borås 2021.

[49] Dao T., Dantigny P. (2011). Control of food spoilage
fungi by ethanol. Food Control.

[50] Appels F. V. W., van den Brandhof J. G., Dijksterhuis J., de Kort G. W., Wösten H. A. B. (2020). Fungal
mycelium classified in different
material families based on glycerol treatment. Commun. Biol.

[51] Paudel S., Regmi S., Janaswamy S. (2023). Effect of glycerol and sorbitol on
cellulose-based biodegradable films. Food Packag.
Shelf Life.

[52] Xiao C., Zhang Z., Zhang J., Lu Y., Zhang L. (2003). Properties
of regenerated cellulose films plasticized with α-monoglycerides. J. Appl. Polym. Sci.

[53] Zhao A., Berglund L., Rosenstock
Völtz L., Swamy R., Antonopoulou I., Xiong S., Mouzon J., Bismarck A., Oksman K. (2025). Fungal Innovation:
Harnessing Mushrooms for Production
of Sustainable Functional Materials. Adv. Funct.
Mater.

[54] Appels F. V. W., Camere S., Montalti M., Karana E., Jansen K. M. B., Dijksterhuis J., Krijgsheld P., Wösten H. A. B. (2019). Fabrication
factors influencing mechanical, moisture- and water-related properties
of mycelium-based composites. Mater. Des.

[55] Peng L., Yi J., Yang X., Xie J., Chen C. (2023). Development and characterization
of mycelium bio-composites by utilization of different agricultural
residual byproducts. J. Bioresour. Bioprod.

[56] Bitting S., Derme T., Lee J., Van Mele T., Dillenburger B., Block P. (2022). Challenges and Opportunities in Scaling up Architectural Applications
of Mycelium-Based Materials with Digital Fabrication. Biomimetics.

[57] Chandra D. K., Kumar A., Mahapatra C. (2025). Advanced Nano-Enhanced
Bioplastics
for Smart Food Packaging: Enhancing Functionalities and Sustainability. Clean. Circ. Bioeconomy.

[58] Sabbah M., Di Pierro P., Cammarota M., Dell’olmo E., Arciello A., Porta R. (2019). Development and properties of new
chitosan-based films plasticized with spermidine and/or glycerol. Food Hydrocolloids.

[59] Joelsson T., Pettersson G., Norgren S., Svedberg A., Höglund H., Engstrand P. (2020). High strength paper from high yield
pulps by means
of hot-pressing. Nord. Pulp Pap. Res. J.

[60] Aiduang W., Jatuwong K., Jinanukul P., Suwannarach N., Kumla J., Thamjaree W., Teeraphantuvat T., Waroonkun T., Oranratmanee R., Lumyong S. (2024). Sustainable Innovation:
Fabrication and Characterization of Mycelium-Based Green Composites
for Modern Interior Materials Using Agro-Industrial Wastes and Different
Species of Fungi. Polymers.

[61] Wang Y., Chang Y., Yu L., Zhang C., Xu X., Xue Y., Li Z., Xue C. (2013). Crystalline structure and thermal
property characterization of chitin from Antarctic krill (Euphausia
superba). Carbohydr. Polym.

[62] Pizzoli M., Ceccorulli G., Scandola M. (1991). Molecular motions of chitosan in
the solid state. Carbohydr. Res.

[63] Nam Y. S., Park W. H., Ihm D., Hudson S. M. (2010). Effect of the degree
of deacetylation on the thermal decomposition of chitin and chitosan
nanofibers. Carbohydr. Polym.

[64] Iqbal M. S., Akbar J., Saghir S., Karim A., Koschella A., Heinze T., Sher M. (2011). Thermal studies
of plant carbohydrate
polymer hydrogels. Carbohydr. Polym.

[65] Sun W., Tajvidi M., Hunt C. G., Howell C. (2021). All-natural smart mycelium
surface with tunable wettability. ACS Appl.
Bio Mater..

[66] Chandra D. K., Kumar A., Mahapatra C. (2024). Fabricating chitosan reinforced biodegradable
bioplastics from plant extract with nature inspired topology. Waste Biomass Valorization.

[67] Zhang M., Zhang Z., Zhang R., Peng Y., Wang M., Cao J. (2023). Lightweight, thermal
insulation, hydrophobic mycelium composites
with hierarchical porous structure: Design, manufacture and applications. Composites, Part B.

